# A Clinical Reasoning Tool for Virtual Patients: Design-Based Research Study

**DOI:** 10.2196/mededu.8100

**Published:** 2017-11-02

**Authors:** Inga Hege, Andrzej A Kononowicz, Martin Adler

**Affiliations:** ^1^ Institute for Medical Education, University Hospital of LMU Munich Muenchen Germany; ^2^ Department of Bioinformatics and Telemedicine Jagiellonian University Medical College Krakow Poland; ^3^ Instruct AG Muenchen Germany

**Keywords:** learning, educational technology, computer-assisted instruction, clinical decision-making

## Abstract

**Background:**

Clinical reasoning is a fundamental process medical students have to learn during and after medical school. Virtual patients (VP) are a technology-enhanced learning method to teach clinical reasoning. However, VP systems do not exploit their full potential concerning the clinical reasoning process; for example, most systems focus on the outcome and less on the process of clinical reasoning.

**Objectives:**

Keeping our concept grounded in a former qualitative study, we aimed to design and implement a tool to enhance VPs with activities and feedback, which specifically foster the acquisition of clinical reasoning skills.

**Methods:**

We designed the tool by translating elements of a conceptual clinical reasoning learning framework into software requirements. The resulting clinical reasoning tool enables learners to build their patient’s illness script as a concept map when they are working on a VP scenario. The student’s map is compared with the experts’ reasoning at each stage of the VP, which is technically enabled by using Medical Subject Headings, which is a comprehensive controlled vocabulary published by the US National Library of Medicine. The tool is implemented using Web technologies, has an open architecture that enables its integration into various systems through an open application program interface, and is available under a Massachusetts Institute of Technology license.

**Results:**

We conducted usability tests following a think-aloud protocol and a pilot field study with maps created by 64 medical students. The results show that learners interact with the tool but create less nodes and connections in the concept map than an expert. Further research and usability tests are required to analyze the reasons.

**Conclusions:**

The presented tool is a versatile, systematically developed software component that specifically supports the clinical reasoning skills acquisition. It can be plugged into VP systems or used as stand-alone software in other teaching scenarios. The modular design allows an extension with new feedback mechanisms and learning analytics algorithms.

## Introduction

In the context of health care education, virtual patients (VPs) are often described as interactive, computer-based programs that simulate real-life clinical encounters [1]. The technical basis of VPs ranges from low-interactive Web pages to high-fidelity simulations or virtual reality scenarios. In the form of interactive patient scenarios, they are typically used to foster clinical reasoning skills acquisition in health care education [2,3]. Interactive patient scenarios are Web-based applications in which a learner navigates through a VP scenario and interacts with the VP in form of menus, questions, or decision points. A variety of commercial and open-source VP systems, such as CASUS, OpenLabyrinth, or i-Human are available and applied in health care education [4]. Such systems provide tools for educators to create VP scenarios and deliver them to their students.

Clinical reasoning or clinical decision making encompasses the application of knowledge to collect and integrate information from various sources to arrive at a diagnosis and a management plan. It is a fundamental skill health care students have to acquire during and after their education. In addition to traditional teaching methods, VPs offer a safe environment to practice clinical reasoning without harming a patient and to prepare learners for clerkships or bedside teaching [2].

However, how clinical reasoning is implemented in VPs varies greatly, and the effect of these design variations on learning outcomes is not yet fully understood [5]. Feedback and scoring are often implemented quantitatively, are outcome-oriented, and do not account for the nonlinear nature [6] of the clinical reasoning process. More process-oriented approaches, such as a study described by Pennaforte et al [7], often require an instructor to be present, thus, limiting the scalability of VPs. Additionally, VP systems do not exploit their full potential concerning the clinical reasoning process. For example, dealing with cognitive errors, explicit development of illness scripts [8], or pattern recognition approaches is rarely implemented in VP systems.

Therefore, our aim was to develop a software tool that can be combined with VP systems, specifically supports clinical reasoning skills acquisition, and assesses all steps of this complex process. We will describe the main components of the software and results of usability tests and a pilot study.

## Methods

### Concept Development

The concept of the tool is based on a grounded theory study, which is an exploratory qualitative research methodology aiming at understanding a phenomenon and developing a theory grounded in the data [9]. We explored the process of learning clinical reasoning based on data resources such as scientific literature or teaching material [10]. The result of the study was an application-oriented framework with five main categories: psychological theories, patient-centeredness, teaching and assessment, learner-centeredness, and context. Each category includes subthemes, such as illness scripts, cognitive errors, self-regulated learning, learning analytics, or cognitive load. This framework served as a basis for developing the concept for the software. We discussed the framework and conclusions on how to transfer it to VPs with health care professionals, educators, and students, and on the basis of these discussions, we developed the functional software requirements (Table 1).

Some of the subthemes of the framework, such as communication, emotions, or authenticity, are related to the design of the VP itself, rather than to the clinical reasoning tool, so they were not translated into software requirements. However, these aspects are important for the VP design process and need to be considered and aligned with the tool.

### Design of the User Interface

**Figure 1:**
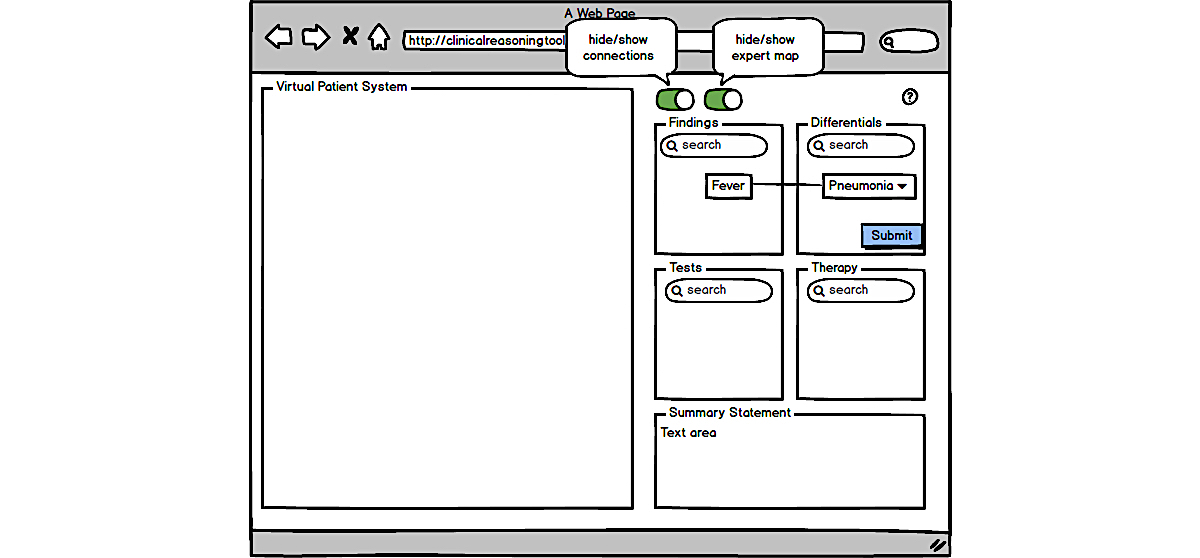
Wireframe model of the clinical reasoning tool (right side) integrated into a virtual patient system (left side).

Figure 1 shows a wireframe model of the clinical reasoning tool with its main components.

For each category (ie, findings, differential diagnoses, tests, and therapies), the learners can search for a term, and either select one from the type-ahead list, which is based on Medical Subject Heading (MeSH) published by the US National Library of Medicine [12], or choose to enter their own entry. Also, negations can be entered, to add negative findings, such as “no fever.”

Differential diagnoses can be marked as “must-not-miss” or as “unlikely/ruled-out” by selecting the option from a context menu. Once the learner has entered a differential diagnosis, the button for submitting a final diagnosis will be activated. After clicking this button, the learner can select one or more diagnoses from his or her differentials and submit them as final diagnoses.

All added nodes (findings, differentials, tests, and therapies) can be deleted, moved within the box, and connected with each other via drag&drop. For example, if a finding speaks against or confirms a diagnosis, the learner can connect the finding with that diagnosis. By clicking on the connection, its color (=weight) and meaning can be changed from red—“speaks against”—to dark blue—“highly related.” Currently, 5 different weights/colors can be assigned to a connection. Thus, learners build their patients’ illness script in the form of a concept map in a step-by-step approach.

Finally, the learner’s task is to compose a short summary statement, usually 2 to 3 sentences about the VP in a text area at the bottom of the tool’s panel. Such a summary statement is a mental abstraction to transform relevant patient-specific details into abstract terms, preferably using semantic qualifiers [13]. This transformation is a crucial step in the clinical reasoning process.

With the 2 switch buttons on top, the learner can toggle the display of connections and can anytime access an expert’s map.

**Table 1:** Overview of categories and subthemes, which have been translated into software requirements and how they have been implemented in the clinical reasoning tool.

Category	Subtheme	Requirements
Psychological theories	Patient illness script	The concept of developing an illness script is implemented as a concept map (directed weighted graph), with findings, differential diagnoses, tests, and therapy options as nodes. Relations can be visualized with connections between the nodes, which can be weighted (eg, “slightly related,” “highly related”)
	Dual processing	Learners can submit a final diagnosis anytime in the virtual patient (VP) scenario to encourage pattern recognition approaches.
Patient-centeredness	Cognitive errors	The final diagnosis/-es of the learner are compared with the expert’s diagnoses. In case of a mismatch, the tool analyzes potential sources of errors or biases.
Teaching/assessment	Methods	Concept mapping as a suitable method of teaching and assessing clinical reasoning is the basis of the tool.
	Scoring	The nodes of the concept map are based on the Medical Subject Heading thesaurus; therefore, they can be scored by comparing them with expert nodes, including synonyms and more/less specific entries.
Learner-centeredness	Learning analytics	After each VP session, the learners can access a dashboard with their clustered scores, development of their performance over time/VPs, and comparison with their peers.
	Feedback	Both, process- and outcome-oriented feedback is provided by the tool and can be accessed by the learner anytime.
Context	Cognitive load	In the development process, we conducted usability tests to test the general usability of the tool and specifically uncover potential improvements in terms of extraneous cognitive load [11].

### Technical Approach

The tool is programmed in Oracle Java, using Java Server Faces as a framework; Hibernate, an open-source Object Relational Mapping solution, for Java applications; and JGraphT, which provides mathematical graph-theory objects and algorithms. All user actions, including a time stamp and at which stage in the VP scenario they were performed, are stored in an Oracle database, but alternative database management systems such as MySQL can be used as well. The client side is implemented in dynamic hypertext markup language, including open source libraries and frameworks such as JQuery, JSPlumb, and D3.js.

The tool is available in English, German, and Polish and can be downloaded under a Massachusetts Institute of Technology license [14]. Exemplary VPs are available in the VP system CASUS [15].

### Patient Illness Script Modeled as a Concept Map

Concept mapping is an approach applied in medical education in general [16] and in clinical reasoning training and assessment [17,18]. In the grounded theory study, which was the basis for the development of the tool, concept mapping was identified as a suitable method of teaching and assessing clinical reasoning skills [10], as it reflects the nonlinear aspects of the process.

**Figure 2:**
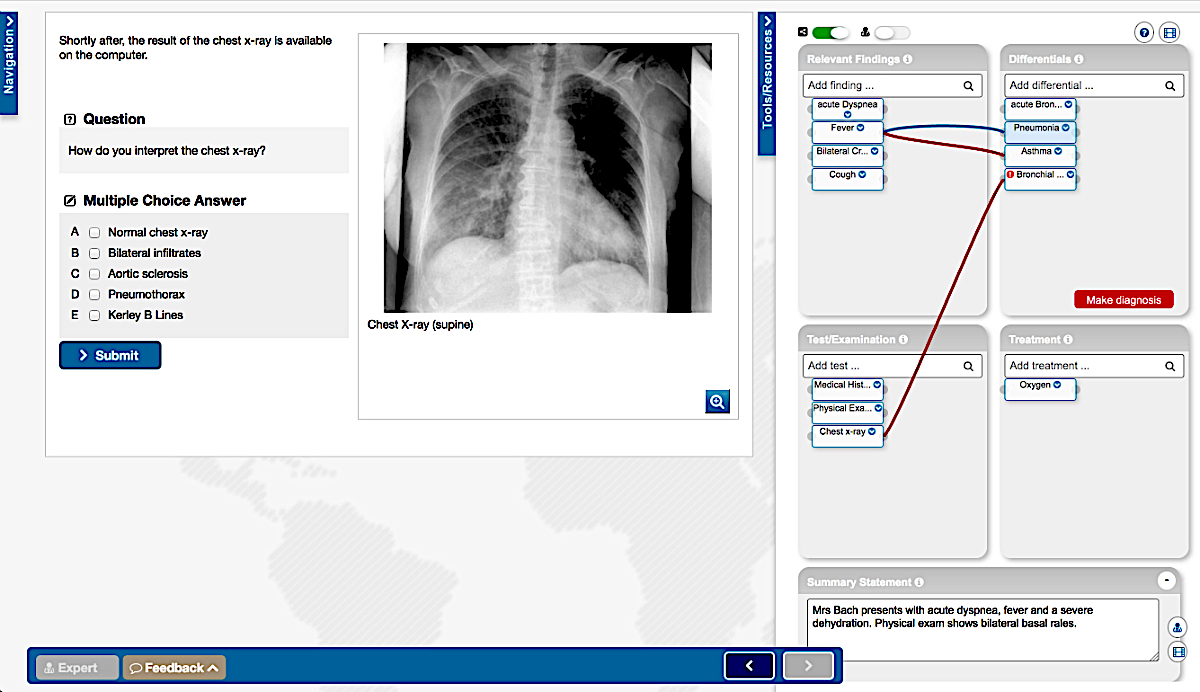
Screenshot of an exemplary VP and a learner's map embedded in the VP system CASUS. The switches on top allow to show/hide all connections and the expert's map; a help page and a short introductory video are available. Diagnoses can be marked as final or working diagnoses and as must-not-miss (exclamation mark) diagnosis.

Illness scripts are mental representations, which link clinical information about a disease, examples of that disease, and its symptoms [8]. Illness scripts are developed by experiencing many different patient cases. The tool uncovers the patient’s illness script and enables the learners to build their own script in the form of a concept map during a VP scenario. Learners can select and connect elements of the concept map and label the connections (Figure 2). In the back end, the concept map is implemented as a directed weighted graph representation of the learner’s and the expert’s maps.

### Dual Processing and Cognitive Errors

Dual processing is the application of analytical and nonanalytical reasoning [19]. Cognitive errors and biases are associated with both approaches [20] and are an essential component of the clinical reasoning process. We considered it as important to allow and encourage the application of both approaches when learners are working with a VP. Therefore, throughout a VP scenario, learners can submit differential diagnoses as their working or final diagnoses and assess their level of confidence with that decision on a slider (scale from 1=“not at all confident” to 100=“very confident”). If there is a mismatch between the learner’s and the expert’s decisions, the software analyzes potential cognitive errors based on the stage, identified findings, differentials, and VPs the learner has accessed previously. The analysis currently focuses on identifying and elaborating 5 common types of cognitive errors—premature closure, availability bias, confirmation bias, representativeness, and base rate neglects [20] (Table 2). To detect base rate neglect and representativeness errors, the experts have to provide additional information, such as disease prevalence, with their concept map.

**Figure 3:**
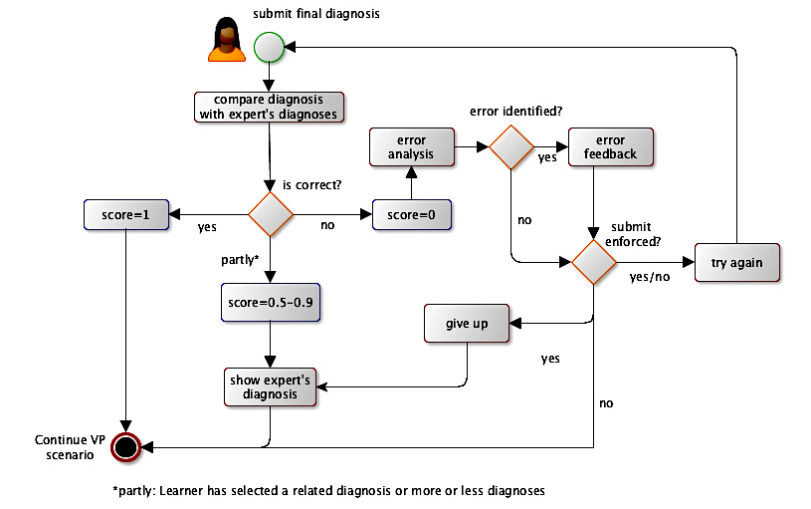
Flowchart of the process of submitting a final diagnosis by a learner.

The clinical reasoning tool then provides feedback and explanations about the error, and the user can choose to try again, continue the VP scenario, or get more feedback (Figure 3).

### Scoring

Scoring and feedback are based on the process of building the concept map and comparing it with an expert’s map.

Partial scores for the final diagnosis submission range between 0.5 and 0.9 (Figure 3), depending on the distance (ie, number of edges) of the learner’s diagnosis to that of the expert’s in the MeSH tree. The distance can be negative if the student’s final diagnosis is more specific than the expert’s solution. For example, if the learner has submitted the final diagnosis as “bacterial pneumonia” and the expert has submitted “pneumonia,” the distance between those two terms in the MeSH hierarchy is −1. The score is then calculated by a heuristic formula:

Score = 1 − (Math. *abs* (distance) / 10)

All changes to the concept map at each stage of the VP scenario are recorded, stored in a database, and scored in comparison with the expert’s map. Because the elements of the map are based on MeSH, we can account for synonyms or more/less specific terms for scoring. Additionally, when the learner moves to the next stage in the VP scenario, all nodes in each category are scored based on the expert’s map at this stage. The heuristic algorithm is as follows:

 Overall score at stage = all scores / (correct nodes + missed nodes) − 0.05 × addNodes

**Table 2:** Overview of errors that can be detected by the tool in case the learner has submitted a final diagnosis that is different from that of the expert’s.

Type of error	Detection	Data required
Premature closure (accepting a diagnosis before it is fully confirmed)	Submission of a final diagnosis at an early stage, after which the expert has added finding(s) or tests that are connected to the final diagnosis	Findings and tests of the learner and the expert (including stage)
		Connections to final diagnosis of expert
		Submission stage
Availability bias (what recently has been seen is more likely to be diagnosed later on)	Learner has worked on or accessed a virtual patient with a related final diagnosis (one Medical Subject Heading hierarchy level up/down) within the last 5 days	Previously created concept maps (date of last access and final diagnoses)
Confirmation bias (tendency to look for confirming evidence for a diagnosis)	Learner has not added disconfirming finding(s) or “speaks against” connections between disconfirming finding and the final diagnosis	Findings of the learner and the expert
		Connections between findings and differential diagnoses
Representativeness (focus on prototypical features of a disease)	Learner has connected nonprototypical findings as “speak against” findings to the correct final diagnosis	Findings of the learner and the expert
		Nonprototypical findings (additional information in expert map)
Base rate neglect (ignoring the true rate of a disease)	A rare final diagnosis has been submitted instead of the more prevalent correct final diagnosis	Differential diagnoses of the learner and the expert
		Prevalence of diagnoses (additional information in expert map)

(all scores=sum of all scores of the user; correct nodes=all nodes scored ≥0.5; missed nodes=nodes added by the expert, but not by the learner at the given stage; addNodes=nodes added by the learner but not present in the expert map).

The learner’s problem representation (summary statement) is scored based on a comparison with the expert’s statement and a list of semantic qualifiers (eg, “acute” vs “chronic”) suggested by Connell et al [21].

The current rating algorithm counts the semantic qualifiers used by the learner and the expert. On the basis of the assessment rubric suggested by Smith et al [22], the score for the use of semantic qualifiers is defined as follows:

- Score 0: Less than 30% of semantic qualifiers used by the expert
- Score 1: <60% and ≥30% of semantic qualifiers used by the expert
- Score 2: ≥60% of semantic qualifiers used by the expert

The weighting of scores is based on the postencounter form scoring model suggested by Durning et al [23].

### Learning Analytics, Feedback, and Adaptability

All scores are clustered based on a model of the clinical reasoning process developed by Charlin et al [24] and correspond with the concept map elements (Table 3). The scores are presented in a student-centered dashboard after a VP scenario has been completed.

Additionally, we implemented clusters for self-directed learning and dual processing, which are not yet fed back to the learner.

The self-directed learning cluster is currently based on the percentage of nodes and connections that have been added by the learner before/without consulting the expert solution. Dual processing considers at which stage a learner submits a final diagnosis; that is, submitting a final diagnosis at an early stage of the VP scenario is an indicator of a more nonanalytical reasoning approach. In a process-oriented approach, the learners can at any stage consult and compare their map with the expert’s or peers’ maps. The progress of the learner is tracked not only within a VP scenario but also throughout a VP collection; these process data feed the learner’s dashboard, in which clustered scores and peer scores are visualized and recommendations for further activities are displayed.

### Application Program Interface to Virtual Patient Systems

A major technical prerequisite for the implementation was the use of the tool as a plug-in for Web-based VP systems through an open application program interface (API).

The communication between the tool and the VP system is required for (1) the initialization and update during the VP session, (2) the display of performance data, and (3) a search functionality (optional). Further details of the API are available in the GitHub Wiki [25].

For the pilot study, we integrated the clinical reasoning tool into the linear VP system CASUS [15,26], a Web-based application for authoring and delivering case-based learning. A CASUS VP typically presents a patient’s story, from the first introduction to the treatment in about 5 to 15 screen cards with a variable combination of text elements, multimedia, and questions. The clinical reasoning tool is displayed in an iframe in the CASUS application; the performance data and the search functionality are integrated in the CASUS dashboard.

### Usability Testing and Implementation of a Pilot Study

During the development process, we conducted usability tests based on a VP with a prototypical version of the tool; participants were 2 health care students and 2 health care professionals, who were familiar with the concept of VPs. For the usability test, we adapted a freely available VP from the eViP repository [27] and presented it with the prototypical clinical reasoning tool. In total, 4 sessions were held with the same testing scenario by one of the authors (IH) in a “Think aloud” approach [28]; participants were briefed about the VP, the prototype, and its purpose; were asked about their expectations, before they could freely explore the VP and the tool; and were further asked about their reactions. Finally, in a debriefing, participants were invited to elaborate on their impressions and suggestions for changes. All findings were documented in field notes and subsequently discussed among the authors. Similar structured follow-up sessions with the same participants were held with a more advanced version of the tool in the VP system CASUS.

From October to December 2016, we implemented a pilot field study with an evaluation of the tool based on 3 VPs in the VP system CASUS. The VPs were reviewed by a course instructor, who regarded the level of difficulty as appropriate for the learners’ level of expertise and confirmed that the VPs match the curricular objectives.

The VPs were integrated into the VP collection of the internal medicine/surgery course at the medical faculty of Ludwig-Maximilian University of Munich (LMU Munich), Germany. The 3 VPs covered the following topics:

- VP 1: a 19-year-old patient with a sore throat; final diagnosis: mononucleosis
- VP 2: a 66-year-old patient with a syncope; final diagnosis: bronchial carcinoma
- VP 3: a 76-year-old patient with acute dyspnea; final diagnosis: pulmonary embolism

In total, 107 fourth year medical students were offered to participate in the study as part of their regular curricular activities. To evaluate the usability of the tool and the integration into the VP system, we used a 5-item questionnaire (Table 4), based on selected questions of the System Usability Scale [29]. The Web-based questionnaire was accessible after each VP session. Participation was voluntary and anonymous.

**Table 3:** Description of clusters on which the learning analytics dashboard is based on.

Concepts in the model by Charlin et al	Cluster
Representation of the problem and determination of objectives of encounter	Scores for adding problems/findings
Investigations	Scores for adding tests
Therapeutic interventions	Scores for adding therapeutic options
Categorization for the purpose of action	Scores for generating differential diagnoses and scores for the final diagnosis
Final representation of the problem and semantic transformation	Scores for the summary statement

**Table 4:** Results of the usability questionnaire (n=10), rated on a 6-point Likert scale (0=totally disagree, 5=totally agree).

Question	Mean response (minimum; maximum)
1. I think that I would like to use the clinical reasoning tool frequently.	3 (0; 5)
2. I found the clinical reasoning tool unnecessarily complex.	3.2 (1; 5)
3. I found the various functions in the clinical reasoning tool were well integrated.	3.4 (2; 5)
4. The clinical reasoning tool helps structuring my thoughts.	2.8 (1; 5)
5. What was good? What should be improved?	3 free text responses

### Ethics Approval and Consent to Participate

The implementation of the pilot study and evaluation was approved by the ethical committee at LMU Munich, Germany.

## Results

### Usability Tests

The prototype-based usability testing revealed some important usability issues; for example, in the prototype, the concept map elements representing the illness script were displayed in a tab layout, thus, unintentionally suggesting an order in which the components had to be worked on. On the basis of this finding, we changed the layout, so that all components were visible at once. Also, two of the participants wanted to enter a negative finding (“no fever”), which was not possible at that time, but was implemented into the next version of the tool. In the follow-up usability tests with a prefinal version of the tool, we identified minor issues, such as the display size and content of tooltips and unclear labeling of buttons. These issues were fixed before the start of the pilot study. The complete usability scenario, the field notes, and a list of the detected and solved issues can be provided on request.

### Pilot Study

During the pilot field testing period from October 15, 2016 to January 31, 2017, with the 3 VPs, 64 of the 107 students created 118 concept maps of varying complexity. This response rate is comparable with similar VP integration scenarios [30]. During the testing period, we constantly evaluated the usage data and further developed the tool. For example, we noted at the beginning of the pilot testing that learners hesitated to interact with the tool; therefore, we further expanded and improved the scaffolding and prompting. Overall, the learners entered 284 problems, 324 differential diagnoses, 158 tests, and 21 treatment options, and submitted 65 final diagnoses; however, only 36 connections were drawn and 19 summary statements composed. Table 5 shows the distribution over the 3 VPs. The questionnaire was completed by 10 participants (Table 4); no usability issues were reported.

Of the free text responses, 2 reported a technical glitch, which was fixed immediately; the 3rd response explicitly liked the idea of the clinical reasoning tool.

**Table 5:** Total number and average number of nodes added per virtual patient (VP) by the users. The number of nodes added by the expert for each VP is shown in parentheses.

Category	Total VP 1	Average VP 1 user (expert)	Total VP 2	Average VP 2 user (expert)	Total VP 3	Average VP 3 user (expert)
Created maps	62		24		31	
Final diagnosis submitted	38 (61%)		7 (29%)		20 (65%)	
Findings/problems	159	2.6 (8)	66	2.8 (7)	59	1.9 (8)
Differential diagnoses	163	2.6 (8)	94	3.9 (8)	67	2.2 (5)
Tests	67	1.1 (5)	50	2.1 (8)	41	1.3 (8)
Therapies	9	0.1 (1)	4	0.2 (1)	8	0.3 (4)
Connections	21	0.3 (5)	14	0.6 (8)	1	0 (5)

## Discussion

### Overview

On the basis of a previous grounded theory exploration [10], our aim was to develop a tool that supports the training of clinical reasoning skills by addressing the most important steps in the clinical reasoning process. The current version of the tool is a good starting point from which we will continue a cyclic process of further evaluation, adaption, and analysis of research studies to advance the functionalities.

The major contribution of our study is a description of an elaborated clinical reasoning tool based on a qualitative research study [10]. Thus, the tool reflects the current research in clinical reasoning training by translating the outcomes of the study into concrete software components and instructional processes.

Concept mapping as the fundamental principle of the tool has been shown to be an effective teaching and assessment approach in health care education (eg, [31,32]). We adapted the typically unstructured approach of concept mapping by providing four main components of clinical reasoning in which the learner can add nodes: problems, differential diagnoses, tests, and therapies. Thus, the steps of the clinical reasoning process and components of the patient illness script are explicitly represented in the maps to guide the learners when they are working on a VP scenario. If learners require further support, they can consult an expert’s concept map and compare it with their own map.

### Pilot Study

The results of the pilot study show that learners interact with the tool, but the average number of nodes added by the learners when compared with the expert map was quite low. Potential explanations could be technical barriers, lack of motivation, or limited clinical reasoning abilities. Because we tried to identify any potential technical barriers with the initial usability tests, we did not receive any support requests by the learners during the pilot study, and an analysis of log files and database entries did not reveal any relevant issues, we believe that technical barriers were not the main reason for the low number of node addition. In 2 of the 3 VPs in more than 60% (n=38) of the VP sessions, the learners submitted a final diagnosis, despite the low number of nodes added, which could indicate a tendency of learners to focus on the outcome (ie, final diagnosis) rather than on the process of clinical reasoning. The participants of the pilot study were students at LMU Munich, who were familiar with VPs since their preclinical years. However, the VPs earlier used by the students were less demanding concerning the clinical reasoning process. Problems and findings of the patient, differential diagnoses, and the final diagnosis were either directly presented in an elaborated way by the VP author, or students had to select appropriate choices from a short list. This simplified approach put the learners in a more passive role and did not emphasize the importance of the process, but the outcome could have influenced students’ interaction with the new tool.

Interestingly, on average, learners added slightly more problems and differential diagnoses for VP 2, but only 29% submitted a final diagnosis. This could indicate that VP 2 was more difficult to solve than VP 1 and VP 3, which is also supported by the higher average number of differential diagnoses added for these maps. A follow-up study is necessary to further investigate the potential effect of VP difficulty on the clinical reasoning process.

Connections between the nodes are substantial components of meaningful concept maps and show that learners understood the concepts and their relations [18]. In the pilot study, only a few connections were drawn, and in the questionnaire, we saw a tendency that the tool did not optimally support learners to structure their thoughts. This might indicate a need for further explanations of concept mapping and/or improvement of the functionality. Further data collection and analysis are needed to find out more about these aspects.

For the pilot study, we combined the tool with a type of a VP, in which the patient is represented in a textual description and multimedia elements. However, the tool can also be integrated into scenarios that represent the patient more authentically and in which more emphasis can be laid on emotions of a patient and identification of problems by actively asking questions. Examples are VPs in the format of conversational agents in which the learner can communicate in natural language with a VP [33] or virtual reality applications [34]. We envision that the tool could also be used in bedside teaching scenarios—for example, as follow-up activities after a patient encounter to help students document their reasoning process and to discuss it with their supervisor. However, it is important to keep in mind that authenticity has to be balanced with both cognitive load and level of expertise of the learner [35]. Thus, less authentic VPs as used in the pilot study can be helpful in preparing novice students for more complex and authentic VP scenarios and real-life patient encounters.

### Further Development

Further development of the tool will focus on implementing machine learning approaches to advance the comparing and scoring of the summary statements and maps.

In the current version of the tool, the learner dashboard is created and displayed within the tool. However, to allow a full integration into learning and teaching infrastructures, such as learning management systems, e-portfolios, or campus management systems, we intend to map the performance data to xAPI [36]. xAPI offers a vocabulary to collect user experiences from different sources and store it in a learning record store.

The open API allows the integration of the clinical reasoning tool into other VP systems than CASUS. Therefore, we are currently working on integrating the tool into the branched VP system OpenLabyrinth [37] as part of the European project WAVES [38].

The tool will also be used for further research studies about clinical reasoning in VPs aiming at answering open questions on the design of a VP to optimally foster the training of clinical reasoning. For example, we are currently implementing a study investigating differences on the reasoning process in undergraduate medical students comparing outcome- and process-oriented expert feedback [39].

Although the response rate of the questionnaire was low, we sense that learners experienced difficulties in structuring their thoughts with the tool, which is exemplified by the very few connections added to the concept maps. The tool was designed based on the results of a qualitative study on the clinical reasoning learning process and VPs [10], and students were involved in all relevant steps in both, the research and the tool implementation process. However, despite these efforts, it seems that the tool does not fully address the learners’ needs; an explanation could be that the students in the pilot study were not familiar with the principles and steps involved in the clinical reasoning process, as this is not explicitly taught at the medical school at LMU Munich. To address this issue, we developed a series of short videos explaining the basic principles of the clinical reasoning process [40], which will be integrated into the tool for the next testing cycle. Additionally, it could be that creating the whole map is too complex for some learners, especially if they are not familiar with this way of thinking. Thus, we are implementing a more adaptive approach in which less advanced learners are guided in a step-wise approach through the map development process, thereby reducing the cognitive load. Depending on the level of expertise and VP difficulty, learners will be prompted to focus on a specific task in the map-creation process. For example, they will be provided with all the nodes and will be asked to focus on the task of creating relevant connections or on the identification of the problems of the patient.

### Limitations

A limitation in our usability testing approach was the low response rate of the survey. This low rate is comparable with the response rates of other VP courses at the medical school at LMU, and we believe that the reason for this is survey fatigue of the participating students; especially in the 4th year, students are exposed to a large number of questionnaires. Furthermore, because of the fact that we only used a subset of the 10-item questionnaire, we are only able to detect usability trends. Our intention was to achieve a higher response rate with a short questionnaire, which turned out to be ineffective. Therefore, we will continue further usability cycles with the full 10-item usability questionnaire in future usage scenarios and studies to collect more reliable and standardized data.

### Conclusions

We believe that the clinical reasoning tool is a valuable addition for Web-based VP systems; it specifically aims to support the clinical reasoning process and includes aspects so far not systematically included in VP systems. We recommend combining the tool with short and carefully designed VPs to make full use of it (see examples at [15]). Additionally, the tool can be used independent from VPs in face-to-face teaching scenarios—for example, to complement clinical reasoning curricula, problem-based-learning seminars, or bedside teaching. We believe that the outcome of our study is relevant for educators and researchers interested in advancing the teaching of clinical reasoning in health care professions.

## Abbreviations

API: application interface
LMU Munich: Ludwig-Maximilian University of Munich
MeSH: Medical Subject Headings
VP: virtual patient
